# Vaccinia virus gene *F3L* encodes an intracellular protein that affects the innate immune response

**DOI:** 10.1099/vir.0.82815-0

**Published:** 2007-07

**Authors:** Graham C. Froggatt, Geoffrey L. Smith, Philippa M. Beard

**Affiliations:** Department of Virology, Faculty of Medicine, Imperial College London, St Mary's Campus, Norfolk Place, London W2 1PG, UK

## Abstract

The Vaccinia virus BTB/kelch protein F3 has been characterized and its effects on virus replication *in vitro* and virus virulence *in vivo* have been determined. The loss of the *F3L* gene had no effect on virus growth, plaque phenotype or cytopathic effect in cell culture under the conditions tested. However, the virulence of a virus lacking *F3L* in an intradermal model was reduced compared with controls, and this was demonstrated by a significantly smaller lesion and alterations to the innate immune response to infection. The predicted molecular mass of the F3 protein is 56 kDa; however, immunoblotting of infected cell lysates using an antibody directed against recombinant F3 revealed two proteins of estimated sizes 37 and 25 kDa.

*Vaccinia virus* (VACV) is a member of the genus *Orthopoxvirus* of the *Poxviridae*; a family of large double-stranded DNA viruses that replicate in the cytoplasm (Moss, 2001). The VACV genome encodes approximately 200 open reading frames (ORFs) (Goebel *et al.*, 1990) with essential genes located mostly in the central, highly conserved region of the genome and non-essential genes in the variable terminal regions (Kotwal & Moss, 1988; Perkus *et al.*, 1991).

Three genes in the VACV genome encode BTB/kelch proteins: *A55R*, *C2L* and *F3L*. These proteins contain an N-terminal BTB (broad-complex, tram-track and bric-a-brac) and a C-terminal kelch domain. The kelch motif sequence is 44–56 aa long, usually occurring as four to seven repeats. Together these form a tertiary structure known as the *β*-propeller, with each repeat unit forming a secondary structure of four anti-parallel *β*-sheets representing a single ‘blade’ of the structure (Adams *et al.*, 2000). The BTB domain mediates protein–protein interactions (Bardwell & Treisman, 1994) and often serves to homodimerize the protein or heterodimerize with other BTB domains. A number of BTB/kelch proteins act as substrate adapters for the ubiquitination machinery, downregulating their target protein and thereby influencing a number of critical cellular pathways (Zhang & Hannink, 2003; Zhang *et al.*, 2004; Angers *et al.*, 2006; Salinas *et al.*, 2006).

Poxviruses are the only viruses known to encode kelch proteins, the number of which varies between species: cowpox virus contains six kelch proteins, ectromelia virus four and monkeypox virus just one. All the kelch proteins in variola virus are missing or fragmented (Shchelkunov *et al.*, 2002). Of the three VACV BTB/kelch proteins, A55 has five kelch repeats, F3 has four and C2 has three.

Previous studies have investigated the phenotype of recombinant VACV strains that lack either the *C2L* or *A55R* genes (Pires de Miranda *et al.*, 2003; Beard *et al.*, 2006). With both these mutants the viral plaque morphology was altered, infected cells produced fewer cellular projections and the characteristic Ca^2+^-independent adhesion of VACV-infected cells was reduced. Murine intradermal infection with either vΔC2 or vΔA55 produced lesions significantly larger than those caused by infection with control viruses. A role for kelch proteins in poxvirus virulence was also suggested by the reports that the sequential deletion of multiple BTB/kelch genes from cowpox virus caused a reduction in virulence (Kochneva *et al.*, 2005), and that one or more BTB/kelch proteins were disrupted in, or lost from, attenuated strains of sheeppox virus, goatpox virus and lumpy skin disease virus (Tulman *et al.*, 2002; Kara *et al.*, 2003).

The goals of this project were to characterize the F3 protein encoded by VACV Western Reserve (WR), specifically to determine its effect on virus growth *in vitro* and virulence *in vivo*.

A recombinant VACV lacking the entire ORF of the *F3L* gene (vΔF3) was generated using transient dominant selection (Falkner & Moss, 1990). The primers pmb21 (5′-CTTAAGTTATTGCATCCACCGAGTGA-3′) and pmb29 (5′-**AGTCAGTCAGTC**CAGTACACAGTATTAACAAATATCG-3′) were used in a PCR to generate a 5′ flanking region, and pmb24 (5′-AAGCTTGCCTTTTAGGGACAGACCAG-3′) and pmb30 (5′-**GACTGACTGACT**TTCATGGAATATAGGGATGGT-3′) were used to generate a 3′ flanking region. The two fragments were then joined by an overlap extension PCR (Horton *et al.*, 1989) (the overlapping complementary DNA regions of primers pmb29 and 30 are shown above in bold) and the resulting fragment was cloned into pSJH7 (Hughes *et al.*, 1991) which contains the *Escherichia coli* guanine xanthine phosphoribosyltransferase (*Ecogpt*) gene as a selectable marker, to create plasmid pPB23. Plasmid pPB23 was transfected into VACV-infected cells, and a deletion and wild-type virus were isolated as described previously (Beard *et al.*, 2006). A revertant virus was constructed using the same method. A 1290 bp PCR product was generated from VACV WR genomic DNA using primers pmb21 and pmb24, comprising full-length *F3L* with flanking regions. This was cloned into pSJH7 to create plasmid pPB25. This was transfected into cells infected with vΔF3 and plaques of the revertant virus (vF3-rev) containing the full-length *F3L* gene were isolated. The genomes of these viruses were analysed by PCR, restriction enzyme digestion and Southern blotting using a probe specific for the *F3L* gene and this confirmed that each virus had the predicted genome structure (data not shown).

The isolation of vΔF3 shows that *F3L* is not essential for virus replication. The growth properties of vΔF3 were analysed and compared with vF3 and vF3-rev by both one-step (m.o.i. of 10) and multi-step (m.o.i. of 0.02) growth curves and no statistical difference was found (data not shown). There was no discernable difference in the morphology of plaques formed by vΔF3 on confluent BS-C-1 cells when compared to vF3 or vF3-rev (Fig. 1a, b[Fig f1]) and the size of the plaques generated was not significantly different on RK-13, BS-C-1, TK^−^143 or CV-1 cell lines (data not shown). vF3, vΔF3 and vF3-rev were used to characterize the effect of F3 on various aspects of VACV-induced cytopathic effect, including the number of VACV-induced cellular projections and relative increase in cell motility (Sanderson *et al.*, 1998), Ca^2+^-dependent adhesion to the extracellular matrix (ECM) (Sanderson & Smith, 1998) and the number of actin tails produced from the cell surface. In each case there was no difference found between vF3, vΔF3 and vF3-rev (data not shown).

The virulence of vΔF3 was examined in both an intranasal (Alcami & Smith, 1992) and intradermal (Tscharke *et al.*, 2002) model of VACV infection. There was no significant difference in weight loss caused by intranasal infection with vΔF3 when compared to vF3 or vF3-rev (5×10^3^ p.f.u. per mouse, data not shown); however, in the intradermal infection model (Fig. 2) vΔF3 produced significantly smaller lesions (*P*<0.05) than both vF3 and vF3-rev from days 6 to 9 post-infection (p.i.) (Fig. 2a[Fig f2]). To determine the basis for this difference in viral virulence, the immune cell populations present in the ears during infection were analysed using flow cytometry as described previously (Jacobs *et al.*, 2006). There was no discernable difference in the proportions of neutrophils, macrophages or CD3 T cells (CD4^+^or CD8^+^). However, in comparison to vF3- and vF3-rev-infected ears, there was a significant increase in the number of natural killer (NK) cells present in vΔF3-infected ears at 4 days p.i. and a significant decrease in the number of T cell receptor (TCR)*γδ* cells at 6 days p.i. (Fig. 2b[Fig f2]). All antibodies used in this study have been described previously (Jacobs *et al.*, 2006). NK cells were defined as the NK1.1^+^ CD3^−^ population using fluorescein isothiocyanate–anti-NK1.1 monoclonal antibody (mAb) (BD Pharmingen) and phycoerythrin (PE)–anti-CD3 mAb (BD Pharmingen). TCR*γδ* cells were labelled with PE–anti-TCR*γδ* mAb (BD Pharmingen).

A rabbit polyclonal antibody to F3 was generated for protein characterization. The C-terminal 310 aa of the F3 protein (including the kelch repeats central region) was amplified from VACV WR by PCR using primers pmb39 (5′-GAATTC**ATG**GATGAGGATTATG-3′) and pmb17 (5′-*AAGCTT*TTATTTACCATCCCATA-3′), generating an *EcoR*I site (underlined) and start site (bold) at the 5′ end of the gene and a *Hin*dIII site at the 3′ end (italics). This product was cloned into pET28(a) (EMD Biosciences) to introduce DNA encoding a his-tag at the 5′ end of the ORF, creating plasmid pPB27. This plasmid was transformed into Rosetta *E. coli* cells (EMD Biosciences) and cultured in Luria–Bertani medium at 37 °C to an OD_600_ of 0.6 before protein expression was induced by the addition of 1 mM isopropyl *β*-d-thiogalactoside for 4 h at 30 °C. The his-tagged recombinant protein was purified from the insoluble fraction of transformed *E. coli* by denaturation of the inclusion body in 6 M guanidine hydrochloride, application of the denatured protein to Ni-NTA beads (Qiagen) and elution of the protein in 0.5 M imidazole. Purified protein was used for rabbit polyclonal antibody production (Harlan Seralabs).

The IgG fraction of the resulting polyclonal serum was used to identify the F3 protein in infected cell lysates (Fig. 3). Confluent BS-C-1 cells were infected at 5 p.f.u. per cell with vF3, vΔF3, vF3-rev or mock-infected either in the presence or absence of the proteasome inhibitor MG132 (10 μM) and the presence or absence of 40 μg cytosine arabinoside (AraC) ml^−1^. Cells were harvested 22 h p.i. and proteins were separated by SDS-PAGE (12 % gel) before being transferred to nitrocellulose and probed with anti-F3 IgG (1 : 1000) or rat mAb p37 directed against F13 (1 : 1000) (Hiller & Weber, 1985). Proteins were visualized using Enhanced Chemiluminescence (ECL) Plus Western blotting detection reagents (Amersham Biosciences) according to the manufacturer's instructions.

The resulting immunoblot showed two proteins of 37 and 25 kDa present in vF3 and vF3-rev-infected cells but absent from vΔF3-infected and mock-infected cells (Fig. 3a, b[Fig f3]). The intensity of both bands was increased by the addition of the proteasomal inhibitor MG132 (Fig. 3c[Fig f3]). The presence of the VACV protein F13 (Fig. 3d[Fig f3]) was used as an infection control. At 22 h p.i., infected cells grown in the presence of DNA replication inhibitor AraC expressed the two F3-specific bands, albeit at lower intensity, indicating that they are the products of early gene expression (Fig. 3e, f[Fig f3]).

Under the conditions tested, immunoblotting did not detect the F3 protein in virions purified by sucrose density-gradient centrifugation (data not shown).

All three BTB/kelch proteins encoded by VACV have now been characterized *in vitro* and *in vivo*. Previous investigations indicated strong similarities between the viruses lacking *C2L* or *A55R*, however, vΔF3 appears to be quite different. Deletion of either *C2L* or *A55R* caused an alteration in viral plaque morphology in which the edges of the plaque appear less distinct than in wild-type and revertant controls (Pires de Miranda *et al.*, 2003; Beard *et al.*, 2006). However, the morphology of vΔF3 plaques is indistinguishable from that of vF3 and vF3-rev (Fig. 1a, b[Fig f1]). Loss of *C2L* and *A55R* each reduced significantly the number of cells that produce projections late during infection and reduced the switch from Ca^2+^-dependent to Ca^2+^-independent ECM interaction. In contrast, vΔF3 has no significant effect on either of these processes. These results provide the first indication that the function of F3 protein during viral infection is distinct from that of either C2 or A55.

The mild attenuation demonstrated by vΔF3 in the early stages of the intradermal infection model (Fig. 2a[Fig f2]) is different from both vΔC2 and vΔA55, which both exhibit an increased lesion size late in infection (Pires de Miranda *et al.*, 2003; Beard *et al.*, 2006). The flow cytometry data presented here indicates a link between F3 and the innate immune response to virus infection. When *F3L* is deleted the percentage of NK cells present in the lesion is increased 4 days p.i. and the percentage of TCR*γδ* cells decreased 2 days later (Fig. 2b[Fig f2]). Both these cell types function as part of the innate immune system (Hamerman *et al.*, 2005; Born *et al.*, 2006) and as such provide a ‘first-line’ defence against viral infection. An increase in the proportion of NK cells early during infection could be responsible for an accelerated immune response and hence earlier decline in the proportion of TCR*γδ* cells observed at day 6 p.i. Notably, the levels of TCR*γδ* cells decline after day 4 in this model (Jacobs *et al.*, 2006). The innate immune response of the skin to VACV infection is of particular interest as intradermal or subcutaneous inoculation is the most commonly used route for administering poxvirus-based vaccines. This environment contains a number of unique immunological features such as specialized *γδ*-expressing T cells known as dendritic epidermal T cells (Hayday & Tigelaar, 2003) and the distinctive antigen presenting cells, Langerhans cells and dermal dendritic cells (Romani *et al.*, 2006). Vaccinia virus is known to express many proteins involved in modulation of innate immune responses of the host, including the production of secreted, soluble decoy receptors and interruption to the intracellular signalling pathways that activate the transcription factor nuclear factor (NF)-*κ*B (Haga & Bowie, 2005). Consequently, the role of F3 in the recruitment and activation of NK cells and TCR*γδ* cells in the skin in response to VACV infection is the subject of further study.

Immunoblotting of infected cell lysates revealed 37 and 25 kDa proteins specific to vF3 and vF3-rev lysates (Fig. 3a[Fig f3]), but the absence of any band attributable to full-length F3 protein (predicted *M*_r_ 56). Further work is under way to identify the provenance of the two bands; the rabbit polyclonal antibody used was raised to a recombinant F3 protein lacking only the N-terminal 20 kDa, and therefore cannot aid in further identification of the fragments. The two polypeptides seen may be derived from a common precursor, or, less likely, might be translated from different RNAs.

Both F3-specific bands are still apparent, at a relatively reduced level, in the presence of AraC, indicating that they are both products of early gene expression. It is also notable that the relative intensity of both bands is increased in the presence of the proteasomal inhibitor MG132, indicating that either they, or factors that regulate the putative F3 cleavage, are targets for proteasomal degradation. Previous work showed that the *A55R* gene encoded a protein of predicted size (Beard *et al.*, 2006) and the level of expression of A55 is not affected by the presence of MG132, emphasizing the differences between these structurally related proteins.

This investigation has shown that the role of the F3 protein in VACV infection is notably different from that of C2 or A55, the other two BTB/kelch proteins encoded by the virus. F3 has no detectable effect on the cytoskeletal organization of the virus-infected cell but does affect the innate immune response to intradermal infection of mice. The mechanisms behind this phenomenon are the subject of future investigations.

## Figures and Tables

**Fig. 1. f1:**
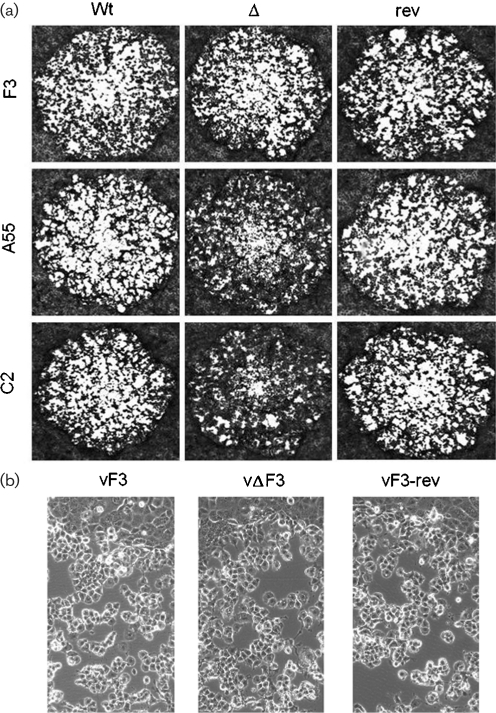
Plaque phenotypes of VACVs lacking each BTB/kelch protein. (a) Plaques produced on confluent BS-C-1 cells by vF3, vΔF3, vF3-rev infection (top row) vA55, vΔA55, vA55-rev (middle row) and vC2, vΔC2 and vC2-rev (bottom row). Infected cells were overlaid with DMEM/2.5 % fetal bovine serum/1.5 % carboxymethylcellulose for 2 days at 37 °C before being stained with crystal violet. (b) Higher magnification detail of plaque edges under phase-contrast microscopy.

**Fig. 2. f2:**
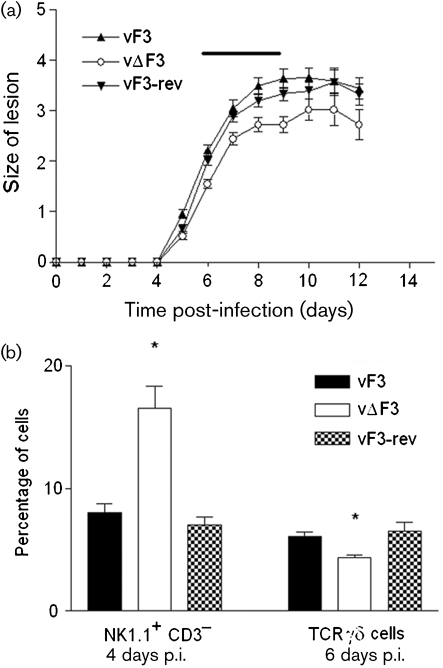
(a) VACV-induced lesions (mm) in C57BL/6 mice infected intradermally with the indicated viruses. The horizontal black line indicates the time points where vΔF3 lesions were significantly smaller than vF3 and vF3-rev. (Student's *t*-test, *P*<0.05). (b) Percentage of NK1.1^+^ CD3^−^ cells present in infected ears at 4 days p.i and TCR*γδ*^+^ cells 6 days p.i. Asterisks indicate significance (Student's *t*-test, *P*<0.05), bars represent mean percentage of cells present (*n*=4)±sem. These data are representative of two separate experiments.

**Fig. 3. f3:**
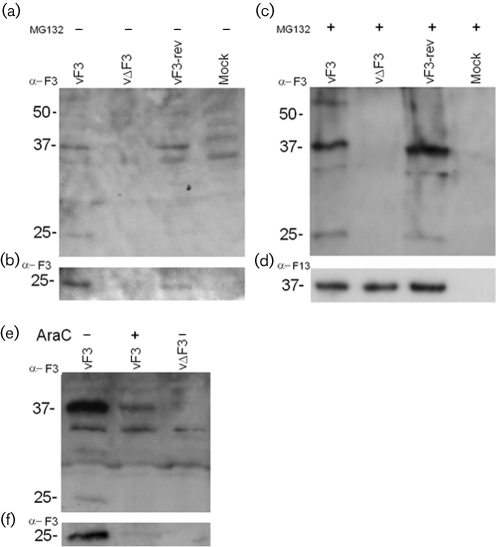
Characterization of the F3 protein. Cells were infected at 5 p.f.u. per cell or mock-infected, with or without MG132 (10 μM), and lysates were analysed by immunoblotting with (a, b) anti-F3 IgG (1 : 1000). (b) Intentionally overdeveloped image of (a), to reveal the 25 kDa band more clearly. (c) Immunoblot of lysates from cells infected with vF3, vΔF3 or vF3-rev in the presence of MG132. (d) Anti-F13 mAb p37 (1 : 1000). (e) AraC (40 μg ml^−1^) was added at time 0 as indicated and the blot was probed with anti-F3 antibody (1 : 1000). (f) Intentionally overexposed image of (e) to reveal the presence of the 25 kDa band.
